# 2-[4-Benzyl-5-(2-fur­yl)-4*H*-1,2,4-triazol-3-ylsulfan­yl]acetamide

**DOI:** 10.1107/S1600536808017170

**Published:** 2008-06-13

**Authors:** Muhammad Zareef, Rashid Iqbal, Muhammad Arfan, Masood Parvez

**Affiliations:** a Department of Chemistry, Quaid-i-Azam University, Islamabad 45320, Pakistan; b Department of Chemistry, The University of Calgary, 2500 University Drive NW, Calgary, Alberta, Canada T2N 1N4

## Abstract

In the title compound, C_15_H_14_N_4_O_2_S, the phenyl ring is inclined at 70.25 (6)° with respect to the approximately planar fur­yl–triazolsulfan­yl–acetamide unit. In the crystal structure, mol­ecules related by inversion centers form dimers *via* inter­molecular N—H⋯O hydrogen bonds between acetamide groups, resulting in eight-membered rings with an *R*_2_^2^(8) motif. In addition, the other H atom of the acetamide group is involved in an inter­molecular hydrogen bond with an N atom of the triazole ring, resulting in chains extended along the *c* axis. The overall effect is the formation of a hydrogen-bonded two-dimensional framework perpendicular to the *a* axis.

## Related literature

For related literature, see: Ahmad *et al.* (2001 ▶); Altman & Solomost (1993 ▶); Bernstein *et al.* (1994 ▶); Chai *et al.* (2003 ▶); Dege *et al.* (2004 ▶); Hashimoto *et al.* (1990 ▶); Kanazawa *et al.* (1988 ▶); Yildirim *et al.* (2004 ▶); Zareef, Iqbal & Parvez (2008 ▶); Zareef, Iqbal, Mirza *et al*. (2008 ▶); Öztürk *et al.* (2004 ▶).
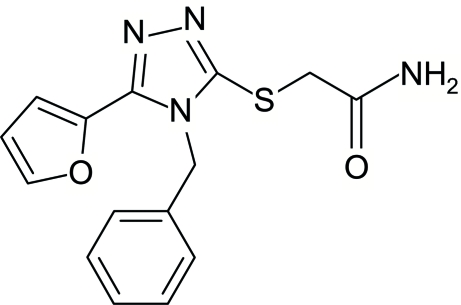


## Experimental

### 

#### Crystal data

C_15_H_14_N_4_O_2_S
*M* *_r_* = 314.36
Monoclinic, 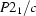

*a* = 15.995 (9) Å
*b* = 7.261 (3) Å
*c* = 13.598 (8) Å
β = 105.46 (2)°
*V* = 1522.1 (14) Å^3^
*Z* = 4
Mo *K*α radiation
μ = 0.23 mm^−1^
*T* = 173 (2) K
0.24 × 0.08 × 0.02 mm

#### Data collection

Nonius KappaCCD diffractometer
Absorption correction: multi-scan (*SORTAV*; Blessing, 1997 ▶) *T*_min_ = 0.948, *T*_max_ = 0.995
5255 measured reflections
3435 independent reflections
2449 reflections with *I* > 2σ(*I*)
*R*_int_ = 0.031

#### Refinement

*R*[*F* ^2^ > 2σ(*F* ^2^)] = 0.045
*wR*(*F* ^2^) = 0.105
*S* = 1.04
3435 reflections
206 parameters
H atoms treated by a mixture of independent and constrained refinement
Δρ_max_ = 0.28 e Å^−3^
Δρ_min_ = −0.26 e Å^−3^

### 

Data collection: *COLLECT* (Hooft, 1998 ▶); cell refinement: *DENZO* (Otwinowski & Minor, 1997 ▶); data reduction: *SCALEPACK* (Otwinowski & Minor, 1997 ▶); program(s) used to solve structure: *SAPI91* (Fan, 1991 ▶); program(s) used to refine structure: *SHELXL97* (Sheldrick, 2008 ▶); molecular graphics: *ORTEP-3 for Windows* (Farrugia, 1997 ▶); software used to prepare material for publication: *SHELXL97*.

## Supplementary Material

Crystal structure: contains datablocks global, I. DOI: 10.1107/S1600536808017170/lh2638sup1.cif

Structure factors: contains datablocks I. DOI: 10.1107/S1600536808017170/lh2638Isup2.hkl

Additional supplementary materials:  crystallographic information; 3D view; checkCIF report

## Figures and Tables

**Table 1:** Hydrogen-bond geometry (Å, °)

*D*—H⋯*A*	*D*—H	H⋯*A*	*D*⋯*A*	*D*—H⋯*A*
N4—H4*A*⋯O2^i^	0.88 (2)	2.01 (2)	2.880 (2)	172 (2)
N4—H4*B*⋯N2^ii^	0.89 (2)	2.01 (2)	2.881 (3)	167 (2)
C9—H9*B*⋯O1	0.99	2.36	3.007 (3)	122

Symmetry codes: (i) 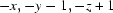; (ii) 
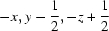
.

**Fig. 1.**
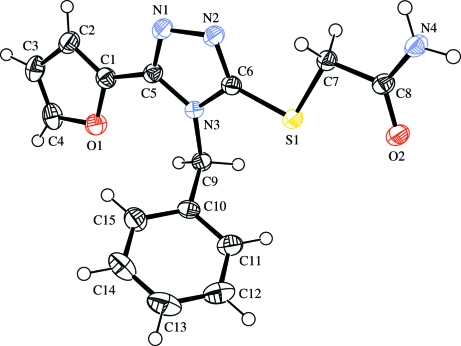
ORTEP-3 (Farrugia, 1997) drawing of the title compound with displacement ellipsoids plotted at 50% probability level.

**Fig. 2.**
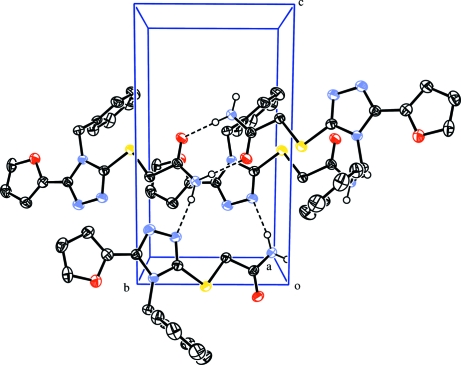
Hydrogen bonding interactions in the unit cell of (I) shown by dashed lines; H-atoms not involved in H-bonds have been omitted.

## supplementary crystallographic information

### Comment

Derivatives of 1,2,4-triazole have significant importance for their broad-spectrum biological and pharmacological activities, such as fungicidal, herbicidal, anticonvulsant, antitumoral, inhibition of cholesterol (Chai *et al.*, 2003; Kanazawa *et al.*, 1988; Hashimoto *et al.*, 1990). In addition, they have many applications in the agriculture domain (Altman & Solomost, 1993). In this paper, we report the synthesis and crystal structure of the title compound, (I).

The structure of the title compound (Fig. 1) is composed of a phenyl ring that is inclined at 70.25 (6)° with respect to a somewhat planar furyl-triazol-thio-acetamide moiety. The mean-planes of the furyl and triazole rings lie at 8.34 (13)° with respect to each other while the atoms in the thioacetamide group (S1/C7/C8/O2/N4) also form a plane which is inclined at 9.48 (10) and 3.45 (12)°, respectively, with furyl and triazole rings. Bond distances and bond angles in (I) agree well with the corresponding bond distances and bond angles reported in compounds closely related to (I) (Zareef, Iqbal & Parvez, 2008; Öztürk *et al.*, 2004; Yildirim *et al.*, 2004; Dege *et al.*, 2004); in all these compounds, the mean-planes of the phenyl rings and the furyl-triazole moieties lie close to right angles. The molecules of (I) lying about inversion centers form dimers as a result of intermolecular N—H···O type hydrogen bonding between acetamide groups; the resulting eight membered rings exhibit an *R*_2_^2^(8)-type motif (Bernstein *et al.*, 1994). The second H-atom of the acetamide group is involved in an intermolecular hydrogen bond with N2 of the triazole ring thus resulting in a chain structure along the *c*-axis. The overall effect is the formation of a hydrogen-bonded two-dimensional framework perpendicular to the a-axis (Fig. 2). The structure is further stabilized by non-classical intramolecular interactions of the type C—H···O (Table 1).

### Experimental

4-Benzyl-1-(2-furoyl)thiosemicarbazide (10 mmol) was dissolved in aqueous 4 N NaOH solution (50 ml). The solution was heated to reflux for 7 h, cooled and filtered. The filtrate was acidified to pH of 4–5, with 4 N HCl. The solid crude product, 4-benzyl-3-(2-furyl)-1*H*-1,2,4-triazole-5(4*H*)-thione, was filtered off, washed with water and recrystallized from aqueous ethanol (60%) (Ahmad *et al.*, 2001). Ethyl S-ester of the triazole was prepared following the procedure reported earlier Zareef, Iqbal, Mirza & *et al.*, 2008). Ethyl-[4-benzyl-5-(2-furyl)-(1,2,4-triazol-3-ylthio)]acetate (10 mmol) was dissolved in dry ethanol (60 ml). Dry ammonia gas was bubbled through the ester solution, with continuous stirring, for 5 hr. The progress of the reaction was monitored by TLC (silica; methanol: chloroform; 1:2). The excess solvent was distilled off under reduced pressure. The crude product was washed with cold water and recrystallized from aqueous ethanol (30%). Crystals of the title compound (I) were grown by slow evaporation of an ethanol solution over 9 days at room temperature (yield 77%).

### Refinement

Though all the H atoms could be distinguished in the difference Fourier map the H-atoms bonded to C-atoms were included at geometrically idealized positions and refined in riding-model approximation with the following constraints: aryl and methylene C—H distances were set to 0.95 and 0.99 Å, respectively; in all these instances *U*_iso_(H) = 1.2 *U*_eq_(C). The H-atoms bonded to N4 were allowed to refine with *U*_iso_(H) = 1.2 *U*_eq_(N4). The final difference map was free of any chemically significant features.

### Crystal data

**Table:**

C_15_H_14_N_4_O_2_S	*F*_000_ = 656
*M**_r_* = 314.36	*D*_x_ = 1.372 Mg m^−^^3^
Monoclinic, *P*2_1_/*c*	Melting point = 417–419 K
Hall symbol: -P 2ybc	Mo *K*α radiation λ = 0.71073 Å
*a* = 15.995 (9) Å	Cell parameters from 5255 reflections
*b* = 7.261 (3) Å	θ = 3.2–27.5º
*c* = 13.598 (8) Å	µ = 0.23 mm^−^^1^
β = 105.46 (2)º	*T* = 173 (2) K
*V* = 1522.1 (14) Å^3^	Plate, colorless
*Z* = 4	0.24 × 0.08 × 0.02 mm

### Data collection

**Table:**

Nonius KappaCCD diffractometer	3435 independent reflections
Radiation source: fine-focus sealed tube	2449 reflections with *I* > 2σ(*I*)
Monochromator: graphite	*R*_int_ = 0.031
*T* = 173(2) K	θ_max_ = 27.5º
ω and φ scans	θ_min_ = 3.2º
Absorption correction: Multi-scan(SORTAV; Blessing, 1997)	*h* = −20→20
*T*_min_ = 0.948, *T*_max_ = 0.995	*k* = −9→7
5255 measured reflections	*l* = −17→17

### Refinement

**Table:**

Refinement on *F*^2^	Hydrogen site location: inferred from neighbouring sites
Least-squares matrix: full	H atoms treated by a mixture of independent and constrained refinement
*R*[*F*^2^ > 2σ(*F*^2^)] = 0.045	*w* = 1/[σ^2^(*F*_o_^2^) + (0.034*P*)^2^ + 0.75*P*] where *P* = (*F*_o_^2^ + 2*F*_c_^2^)/3
*wR*(*F*^2^) = 0.105	(Δ/σ)_max_ < 0.001
*S* = 1.04	Δρ_max_ = 0.28 e Å^−^^3^
3435 reflections	Δρ_min_ = −0.26 e Å^−^^3^
206 parameters	Extinction correction: SHELXL97 (Sheldrick, 2008), Fc^*^=kFc[1+0.001xFc^2^λ^3^/sin(2θ)]^-1/4^
Primary atom site location: structure-invariant direct methods	Extinction coefficient: 0.0052 (16)
Secondary atom site location: difference Fourier map	

### Special details

**Table:**

Geometry. All e.s.d.'s (except the e.s.d. in the dihedral angle between two l.s. planes) are estimated using the full covariance matrix. The cell e.s.d.'s are taken into account individually in the estimation of e.s.d.'s in distances, angles and torsion angles; correlations between e.s.d.'s in cell parameters are only used when they are defined by crystal symmetry. An approximate (isotropic) treatment of cell e.s.d.'s is used for estimating e.s.d.'s involving l.s. planes.
Refinement. Refinement of *F*^2^ against ALL reflections. The weighted *R*-factor *wR* and goodness of fit *S* are based on *F*^2^, conventional *R*-factors *R* are based on *F*, with *F* set to zero for negative *F*^2^. The threshold expression of *F*^2^ > σ(*F*^2^) is used only for calculating *R*-factors(gt) *etc*. and is not relevant to the choice of reflections for refinement. *R*-factors based on *F*^2^ are statistically about twice as large as those based on *F*, and *R*- factors based on ALL data will be even larger.

### Fractional atomic coordinates and isotropic or equivalent isotropic displacement parameters (Å^2^)

**Table:**

	*x*	*y*	*z*	*U*_iso_*/*U*_eq_	
S1	0.12862 (3)	0.04606 (6)	0.49096 (4)	0.02689 (16)	
O1	0.29228 (9)	0.72417 (18)	0.45345 (11)	0.0318 (4)	
O2	0.07346 (10)	−0.29345 (18)	0.53495 (11)	0.0330 (4)	
N1	0.14752 (11)	0.4036 (2)	0.28542 (12)	0.0290 (4)	
N2	0.11490 (11)	0.2402 (2)	0.31391 (12)	0.0287 (4)	
N3	0.19418 (10)	0.3766 (2)	0.45331 (11)	0.0219 (4)	
N4	−0.01125 (12)	−0.3863 (2)	0.38142 (14)	0.0280 (4)	
H4A	−0.0253 (14)	−0.489 (3)	0.4074 (17)	0.034*	
H4B	−0.0375 (14)	−0.358 (3)	0.3169 (18)	0.034*	
C1	0.23596 (13)	0.6584 (3)	0.36615 (16)	0.0279 (5)	
C2	0.22942 (15)	0.7781 (3)	0.28834 (17)	0.0335 (5)	
H2	0.1947	0.7646	0.2202	0.040*	
C3	0.28508 (15)	0.9285 (3)	0.32900 (18)	0.0367 (5)	
H3	0.2950	1.0347	0.2929	0.044*	
C4	0.32076 (15)	0.8915 (3)	0.42773 (18)	0.0355 (5)	
H4	0.3602	0.9699	0.4736	0.043*	
C5	0.19400 (13)	0.4820 (3)	0.36896 (15)	0.0239 (4)	
C6	0.14400 (13)	0.2270 (2)	0.41377 (15)	0.0236 (4)	
C7	0.05176 (13)	−0.0836 (2)	0.39448 (15)	0.0243 (4)	
H7A	−0.0038	−0.0160	0.3718	0.029*	
H7B	0.0747	−0.1049	0.3346	0.029*	
C8	0.03843 (13)	−0.2653 (3)	0.44313 (15)	0.0246 (4)	
C9	0.23541 (13)	0.4072 (3)	0.56249 (14)	0.0250 (4)	
H9A	0.1943	0.3721	0.6023	0.030*	
H9B	0.2486	0.5399	0.5742	0.030*	
C10	0.31828 (13)	0.2975 (3)	0.60034 (14)	0.0250 (4)	
C11	0.32270 (15)	0.1562 (3)	0.66992 (16)	0.0355 (5)	
H11	0.2734	0.1278	0.6934	0.043*	
C12	0.39864 (17)	0.0557 (3)	0.70563 (19)	0.0484 (6)	
H12	0.4012	−0.0407	0.7536	0.058*	
C13	0.47022 (17)	0.0959 (4)	0.6714 (2)	0.0501 (7)	
H13	0.5222	0.0273	0.6959	0.060*	
C14	0.46651 (15)	0.2352 (3)	0.6017 (2)	0.0437 (6)	
H14	0.5159	0.2621	0.5779	0.052*	
C15	0.39075 (14)	0.3365 (3)	0.56616 (17)	0.0344 (5)	
H15	0.3885	0.4329	0.5183	0.041*	

### Atomic displacement parameters (Å^2^)

**Table:**

	*U*^11^	*U*^22^	*U*^33^	*U*^12^	*U*^13^	*U*^23^
S1	0.0291 (3)	0.0266 (3)	0.0229 (3)	−0.0040 (2)	0.0033 (2)	0.0036 (2)
O1	0.0348 (8)	0.0264 (7)	0.0321 (8)	−0.0040 (6)	0.0055 (7)	0.0003 (6)
O2	0.0390 (9)	0.0298 (7)	0.0253 (8)	−0.0067 (7)	0.0000 (7)	0.0067 (6)
N1	0.0328 (10)	0.0305 (8)	0.0239 (9)	−0.0027 (8)	0.0080 (8)	0.0029 (7)
N2	0.0326 (10)	0.0296 (8)	0.0227 (9)	−0.0040 (8)	0.0051 (8)	0.0024 (7)
N3	0.0218 (9)	0.0233 (8)	0.0198 (8)	0.0000 (7)	0.0041 (7)	0.0006 (6)
N4	0.0366 (11)	0.0223 (8)	0.0234 (9)	−0.0018 (8)	0.0050 (8)	0.0033 (7)
C1	0.0263 (11)	0.0291 (10)	0.0292 (11)	0.0009 (9)	0.0093 (9)	−0.0014 (8)
C2	0.0378 (13)	0.0334 (11)	0.0302 (12)	−0.0015 (10)	0.0106 (10)	0.0049 (9)
C3	0.0428 (14)	0.0289 (11)	0.0433 (14)	−0.0009 (10)	0.0203 (11)	0.0070 (10)
C4	0.0368 (13)	0.0225 (10)	0.0505 (15)	−0.0056 (9)	0.0176 (12)	−0.0035 (9)
C5	0.0244 (10)	0.0256 (9)	0.0225 (10)	0.0027 (8)	0.0077 (9)	0.0012 (8)
C6	0.0224 (10)	0.0257 (9)	0.0224 (11)	0.0002 (8)	0.0053 (9)	−0.0011 (8)
C7	0.0263 (11)	0.0229 (9)	0.0230 (10)	−0.0020 (8)	0.0053 (9)	0.0013 (8)
C8	0.0253 (11)	0.0241 (9)	0.0241 (11)	0.0041 (8)	0.0060 (9)	0.0028 (8)
C9	0.0256 (11)	0.0289 (10)	0.0205 (10)	−0.0023 (9)	0.0060 (9)	−0.0022 (8)
C10	0.0251 (11)	0.0272 (10)	0.0200 (10)	−0.0004 (8)	0.0012 (9)	−0.0052 (8)
C11	0.0348 (13)	0.0403 (12)	0.0285 (12)	0.0038 (11)	0.0036 (10)	0.0031 (10)
C12	0.0476 (16)	0.0499 (14)	0.0406 (15)	0.0118 (13)	−0.0004 (12)	0.0127 (11)
C13	0.0333 (14)	0.0514 (14)	0.0559 (17)	0.0104 (12)	−0.0050 (13)	−0.0021 (13)
C14	0.0252 (12)	0.0450 (13)	0.0591 (17)	−0.0014 (11)	0.0081 (12)	−0.0083 (12)
C15	0.0284 (12)	0.0326 (11)	0.0412 (14)	−0.0018 (10)	0.0076 (10)	0.0009 (9)

### Geometric parameters (Å, °)

**Table:**

S1—C6	1.740 (2)	C3—H3	0.9500
S1—C7	1.805 (2)	C4—H4	0.9500
O1—C1	1.371 (2)	C7—C8	1.516 (3)
O1—C4	1.375 (2)	C7—H7A	0.9900
O2—C8	1.242 (2)	C7—H7B	0.9900
N1—C5	1.310 (3)	C9—C10	1.514 (3)
N1—N2	1.392 (2)	C9—H9A	0.9900
N2—C6	1.316 (3)	C9—H9B	0.9900
N3—C6	1.372 (2)	C10—C11	1.385 (3)
N3—C5	1.378 (2)	C10—C15	1.388 (3)
N3—C9	1.472 (2)	C11—C12	1.389 (3)
N4—C8	1.324 (3)	C11—H11	0.9500
N4—H4A	0.88 (2)	C12—C13	1.377 (4)
N4—H4B	0.89 (2)	C12—H12	0.9500
C1—C2	1.351 (3)	C13—C14	1.376 (4)
C1—C5	1.452 (3)	C13—H13	0.9500
C2—C3	1.424 (3)	C14—C15	1.390 (3)
C2—H2	0.9500	C14—H14	0.9500
C3—C4	1.339 (3)	C15—H15	0.9500
			
C6—S1—C7	97.69 (9)	C8—C7—H7B	110.4
C1—O1—C4	105.85 (16)	S1—C7—H7B	110.4
C5—N1—N2	107.29 (16)	H7A—C7—H7B	108.6
C6—N2—N1	107.04 (15)	O2—C8—N4	124.19 (18)
C6—N3—C5	104.00 (16)	O2—C8—C7	120.25 (17)
C6—N3—C9	124.93 (15)	N4—C8—C7	115.56 (17)
C5—N3—C9	131.06 (16)	N3—C9—C10	112.32 (15)
C8—N4—H4A	118.8 (14)	N3—C9—H9A	109.1
C8—N4—H4B	121.1 (14)	C10—C9—H9A	109.1
H4A—N4—H4B	119 (2)	N3—C9—H9B	109.1
C2—C1—O1	110.49 (18)	C10—C9—H9B	109.1
C2—C1—C5	130.4 (2)	H9A—C9—H9B	107.9
O1—C1—C5	119.10 (17)	C11—C10—C15	119.0 (2)
C1—C2—C3	106.2 (2)	C11—C10—C9	120.18 (19)
C1—C2—H2	126.9	C15—C10—C9	120.82 (18)
C3—C2—H2	126.9	C10—C11—C12	120.6 (2)
C4—C3—C2	106.91 (19)	C10—C11—H11	119.7
C4—C3—H3	126.5	C12—C11—H11	119.7
C2—C3—H3	126.5	C13—C12—C11	119.9 (2)
C3—C4—O1	110.53 (19)	C13—C12—H12	120.0
C3—C4—H4	124.7	C11—C12—H12	120.0
O1—C4—H4	124.7	C14—C13—C12	120.1 (2)
N1—C5—N3	110.81 (17)	C14—C13—H13	120.0
N1—C5—C1	121.34 (18)	C12—C13—H13	120.0
N3—C5—C1	127.84 (18)	C13—C14—C15	120.1 (2)
N2—C6—N3	110.85 (16)	C13—C14—H14	119.9
N2—C6—S1	127.53 (15)	C15—C14—H14	119.9
N3—C6—S1	121.56 (14)	C10—C15—C14	120.3 (2)
C8—C7—S1	106.52 (13)	C10—C15—H15	119.9
C8—C7—H7A	110.4	C14—C15—H15	119.9
S1—C7—H7A	110.4		
			
C5—N1—N2—C6	−0.5 (2)	C9—N3—C6—N2	178.80 (17)
C4—O1—C1—C2	0.4 (2)	C5—N3—C6—S1	176.91 (14)
C4—O1—C1—C5	−179.59 (18)	C9—N3—C6—S1	−3.8 (3)
O1—C1—C2—C3	0.1 (2)	C7—S1—C6—N2	−7.9 (2)
C5—C1—C2—C3	−180.0 (2)	C7—S1—C6—N3	175.14 (16)
C1—C2—C3—C4	−0.5 (3)	C6—S1—C7—C8	172.23 (13)
C2—C3—C4—O1	0.7 (3)	S1—C7—C8—O2	4.6 (2)
C1—O1—C4—C3	−0.7 (2)	S1—C7—C8—N4	−175.01 (15)
N2—N1—C5—N3	0.2 (2)	C6—N3—C9—C10	79.9 (2)
N2—N1—C5—C1	−178.64 (17)	C5—N3—C9—C10	−101.0 (2)
C6—N3—C5—N1	0.2 (2)	N3—C9—C10—C11	−113.1 (2)
C9—N3—C5—N1	−179.06 (18)	N3—C9—C10—C15	66.9 (2)
C6—N3—C5—C1	178.91 (19)	C15—C10—C11—C12	0.4 (3)
C9—N3—C5—C1	−0.3 (3)	C9—C10—C11—C12	−179.5 (2)
C2—C1—C5—N1	7.9 (3)	C10—C11—C12—C13	−0.3 (4)
O1—C1—C5—N1	−172.15 (18)	C11—C12—C13—C14	−0.1 (4)
C2—C1—C5—N3	−170.7 (2)	C12—C13—C14—C15	0.4 (4)
O1—C1—C5—N3	9.3 (3)	C11—C10—C15—C14	−0.2 (3)
N1—N2—C6—N3	0.6 (2)	C9—C10—C15—C14	179.81 (19)
N1—N2—C6—S1	−176.61 (15)	C13—C14—C15—C10	−0.2 (3)
C5—N3—C6—N2	−0.5 (2)		

### Hydrogen-bond geometry (Å, °)

**Table:**

*D*—H···*A*	*D*—H	H···*A*	*D*···*A*	*D*—H···*A*
N4—H4A···O2^i^	0.88 (2)	2.01 (2)	2.880 (2)	172 (2)
N4—H4B···N2^ii^	0.89 (2)	2.01 (2)	2.881 (3)	167 (2)
C9—H9B···O1	0.99	2.36	3.007 (3)	122

Symmetry codes: (i) −*x*, −*y*−1, −*z*+1; (ii) −*x*, *y*−1/2, −*z*+1/2.
